# Presentation of Two Cases with Early Extracranial Metastases from Glioblastoma and Review of the Literature

**DOI:** 10.1155/2016/8190950

**Published:** 2016-05-09

**Authors:** Maria Dinche Johansen, Per Rochat, Ian Law, David Scheie, Hans Skovgaard Poulsen, Aida Muhic

**Affiliations:** ^1^Department of Radiation Biology, The Finsen Center, Rigshospitalet, Blegdamsvej 9, 2100 Copenhagen, Denmark; ^2^Department of Neurosurgery, The Neurocenter, Rigshospitalet, Blegdamsvej 9, 2100 Copenhagen, Denmark; ^3^Department of Clinical Physiology, Nuclear Medicine and PET, Center of Diagnostic Investigation, Rigshospitalet, Blegdamsvej 9, 2100 Copenhagen, Denmark; ^4^Department of Pathology, Center of Diagnostic Investigation, Rigshospitalet, Blegdamsvej 9, 2100 Copenhagen, Denmark; ^5^Department of Oncology, The Finsen Center, Rigshospitalet, Blegdamsvej 9, 2100 Copenhagen, Denmark

## Abstract

Extracranial metastases from glioblastoma are rare. We report two patients with extracranial metastases from glioblastoma. Case 1 concerns a 59-year-old woman with multiple metastases that spread early in the course of disease. What makes this case unusual is that the tumor had grown into the falx close to the straight sinus and this might be an explanation to the early and extensive metastases. Case 2 presents a 60-year-old man with liver metastasis found at autopsy, and, in this case, it is more difficult to find an explanation. This patient had two spontaneous intracerebral bleeding incidents and extensive bleeding during acute surgery with tumor removal, which might have induced extracranial seeding. The cases presented might have hematogenous spreading in common as an explanation to extracranial metastases from GBM.

## 1. Introduction

Glioblastoma (GBM) is the most frequent adult primary tumor of the central nervous system with median survival of 14.6 months in patients with newly diagnosed glioblastoma. The majority of patients experience local progression within the central nervous system [1].

Extracranial metastases (ECMs) are uncommon events seen in these patients, and most patients metastasize to only one or two extracranial foci [2]. Most frequent localization of ECM is regional lymph nodes, mostly cervical, lungs, liver, and bone [2, 3]. The rarity of ECM makes epidemiological analysis challenging, but it has been suggested that the median time from diagnosis to detection of ECM is 8.5 months and the time from ECM to death is 1.5 months [2]. A study has found that 20% of GBM patients have circulating tumor cells in peripheral blood, pointing out the ability to escape the central nervous system [4]. This combined with longer survival time should increase the awareness of ECM.

Here, we report two cases that demonstrate the ability of GBM to metastasize in one case to multiple organs simultaneously.

## 2. Case Presentations

### 2.1. Case 1

This case presents a 59-year-old woman with a history of type 2 diabetes, hypertension, and tobacco consumption (20 cigarettes per day for 45 years). The patient visited an ophthalmologist due to three months of blurred vision and two months of headache. This revealed impaired vision (right sided homonymous hemianopsia) and memory and concentration difficulties. Contrast enhanced CT and MRI of the brain showed a 4 × 5 × 4 cm solitaire left sided occipital tumor with midline shift. At this point, helical CT of thorax and abdomen was inconspicuous. The patient was treated with corticosteroids.

**Figure 1:**
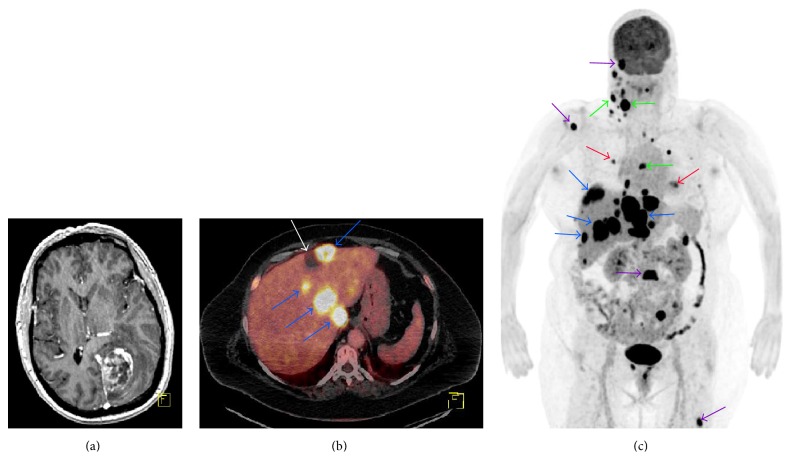
Case  1. (a) Preoperative post-contrast enhanced T1 weighted MRI showing the localization of the tumor in close proximity to the falx. (b) Fused FDG PET/CT scanning at liver level 5 months after diagnosis of GBM showing multiple metabolically active metastases (blue) and inactive liver cyst (white). (c) Frontal maximum intensity projection (MIP) image of whole body FDG PET scanning identifying disseminated metastatic spread to lymph nodes (green), lungs (red), bone (purple), and liver (blue). Physiological excretion to intestines, kidneys, and the bladder.

Two weeks later macro radical tumor resection was achieved using Gliolan® when the patient underwent left sided occipital craniotomy. During surgery, there was copious venous bleeding. Early postoperative post-contrast T1 weighted MRI showed no measurable tumor. Postoperatively the patient suffered from right sided hemianopsia. Histological examination revealed a cellular astrocytic glioma with pleomorphic nuclei, numerous mitoses, microvascular proliferation, pseudopalisading necrosis, and thrombosed vessels. Upon immunohistochemical examination, the tumor cells stained positive for GFAP, p53 (pronounced and strong in almost all tumor cells), map2, and olig2. Immunohistochemical stainings did not reveal IDH1- or ATRX-mutations. Ki67 was high. These findings were compatible with glioblastoma, WHO grade IV. PCR-analysis revealed an average O^6^-methylguanine-DNA methyltransferase (MGMT) promoter methylation of 18%. PET scanning using the radiolabeled amino acid analog O-(2-^18^F-fluoroethyl)-L-tyrosine (FET) performed at radiation treatment planning revealed a few mL of active tissue close to the cortex. The patient received radiotherapy, 2 Gy/5 days per week, for a total dose of 60 Gy with concomitant chemotherapy (temozolomide 75 mg/m^2^ per day) for six weeks. This was followed by adjuvant chemotherapy temozolomide; the first dose was administered at 150 mg/m^2^ for five days and second and third cycles were administered at 200 mg/m^2^ for five days. Routine surveillance MRI from the start of the second series of adjuvant chemotherapy found no sign of tumor recurrence. Clinically, however, the patient complained about circumscribed pain on the right abdominal side and on the right side of her neck. This was examined at a local hospital with ultra sound, whole body FDG PET/CT scanning (Figures 1(b) and 1(c)) and three biopsies of the cervical lymph nodes. There were no signs of local recurrence at the resection cavity on brain MRI.

**Figure 2:**
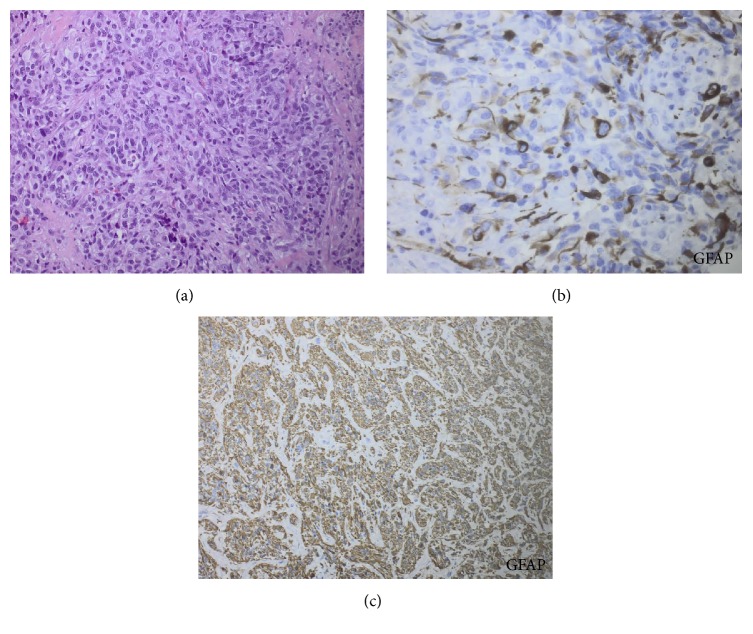
Histopathology from both cases. (a) HE staining (×20) of cervical lymph node metastasis from case 1. (b) GFAP staining (×40) of cervical lymph node metastasis from case 1. (c) GFAP staining (×10) of liver metastasis from case 2.

The biopsies were examined by pathologists at the local hospital and reexamined by neuropathologists who specialize in neurooncology. Microscopic examination revealed pronounced tumor necrosis. Tumor cells were large and epithelioid with vesicular nuclei with prominent nucleoli (Figure 2(a)). Spindled cells were also observed. There were numerous mitoses. The tumor cells stained positive for S-100, vimentin, CD56, and GFAP (Figure 2(b)). There was focal staining for olig2 and synaptophysin, while map2 was almost negative. As in the brain tumor, the cells showed strong and pronounced staining for p53. There were negative stainings for pancytokeratin, CK7, CK20, TTF1, melan A, and CD45. The average MGMT promoter methylation was 2%. The diagnosis was lymph node metastasis from malignant tumor, most likely glioblastoma. The conclusion based on scans and histology was multiple glioblastoma metastases to lymph nodes in cervical and mediastinal region, liver, bones, and both lungs.

Second line treatment with irinotecan (250 mg) and bevacizumab (1000 mg) was initiated, but this was discontinued after one series due to deterioration of the patient's clinical condition.

During this period—from lymph node biopsy till admission to hospice—blood test showed signs of liver damage with elevated and increasing values of lactate dehydrogenase (LDH; 230–1331 U/L [normal range, <205 U/L]), elevated alkaline phosphatase (134–446 U/L [normal range, <105 U/L]), normal to slightly elevated alanine transferase (ALAT; 22–68 U/L [normal range, <45 U/L]), normal bilirubin (6–12 *μ*mol/L [normal range, 5–25 *μ*mol/L]), and decreased lymphocytes (0.26–1.1 × 10^9^ [normal range, 1.0–3.5 × 10^9^]). No further diagnostic investigations were performed out of respect for the patient's wish.

The patient was referred to hospice, where she had a rapid decline with confusion, insufficient nutritional intake, nausea, and increased pain. She died eight months after diagnosis with a clinical pattern of liver insufficiency.

### 2.2. Case 2

The second case presents a 60-year-old man with a history of hypertension. He was brought to the emergency room because of several generalized tonic-clonic seizures, and a head CT showed a frontal intracerebral hemorrhage. In the following weeks, two MRI scans had to be cancelled because the patient suffered from claustrophobia and could not cooperate. Contrast CT scan was not performed, even though it would have been relevant. A cerebral angiography was performed and excluded a vascular cause of the hemorrhage. A frontal tumor was found when the patient had an MRI four months after the initial intracerebral hemorrhage.

The patient suffered from several subsequent seizures. An operation was scheduled for removal of the tumor. The patient was brought to the emergency room a few days before the scheduled surgery with decreasing consciousness, and an acute CT scan revealed a new bleeding from the tumor. He therefore underwent emergency surgery during which there was extensive bleeding during removal of the tumor. The patient had no early postoperative MRI because the hypothesized diagnosis was metastasis from an unknown malignant melanoma. The microscopic examination revealed a malignant astrocytoma with numerous mitoses, microvascular proliferation, and pseudopalisading necrosis. The tumor cells stained positive for GFAP, map2, and olig2. p53 demonstrated weak staining. Immunohistochemical stainings did not reveal IDH1- or ATRX-mutations. Ki67 was high. The findings were compatible with glioblastoma, WHO grade IV. The average MGMT promoter methylation was 42%.

Due to the new intracerebral bleeding, the patient spent one month at an intensive care unit and after this the patient and his family decided not to go through radiation. At this point, the patient was at performance status 4 and was treated at a palliative care unit until his death 10 months after his first hemorrhage. An autopsy was performed, and this revealed well demarcated solid metastasis in the liver. The solid metastasis measured 1 × 1 × 0.5 cm. Microscopic examination revealed a metastasis composed of spindled cells with scattered mitoses. Necrosis or microvascular proliferation was not observed. Immunohistochemical staining revealed strong and uniform staining for GFAP (Figure 2(c)) and S-100 but not IDH1-mutation, and they were negative for map2, olig2, melan A, pancytokeratin, desmin, and actin. P53 staining was weak. These findings were compatible with metastasis from glioblastoma. The MGMT promoter methylation was 37%. There was no suspicion or complaints during the course of the disease that could lead to suspicion of liver metastasis.

## 3. Discussion

Despite the rarity, ECMs from GBM have been known for many years, with the first documented case in 1928 [5]. In 1955, Weiss established diagnostic criteria for extraneural metastases from primary CNS tumors. These included a clinical history of primary CNS tumor, a complete postmortem examination, and histological correlation between the primary CNS tumor and the presumed extraneural metastases [6]. Today, not all new cases have a complete postmortem examination because of new imaging methods—such as PET/CT scans—making it possible to detect a potential undiscovered primary tumor other than GBM.

In a meta-analysis by Lun et al., 83 published cases of ECM from GBM were found in the period from 1928 to 2009 [2]. Increasing incidence of ECM has been suggested but possible explanations are increased interest among specialists, improved access to health care, improved neuroimaging, and advanced multimodal treatment of gliomas [2, 7]. A meta-analysis by Anghileri et al. supports the idea that prolonged survival of GBM patients is associated with greater risk of ECM and it is emphasized that this finding does not rule out the hypothesis that GBM subclones contribute to tumor cell dissemination [4, 8].

The rarity of GBM may be due to a number of factors: the preference of GBM cells to adhere to neural stroma, the low number of circulating GBM cells compared to the number of circulating monocytes, and the need for a metastatic niche in distant organs in order for GBM cells to establish a metastasis [2].

Most frequent localization of ECM is regional lymph nodes, mostly cervical, lungs, liver, and bone [3]. Although ECMs are mostly seen in patients with preceding intracranial surgery, such as ventriculoperitoneal shunt [9], ECM in the absence of previous surgery has been described [10]. In most previously published cases, the tumor metastasized to either only one or two extracranial organs or the time from diagnosis to detection of ECM was more than 5 months [2]. In their meta-analysis, Lun et al. suggest that it may be more difficult to detect neck and liver metastasis as fast as metastasis in other areas [2].

So far, no standard treatment for ECM exists. This might be because the patients are already in the late stage of the disease when the metastases are discovered, and at that point only palliative care is needed. Ray et al. suggest organ-specific considerations for patients with ECM and that the oncological treatment focuses on systemic chemotherapy [11].

Our case 1 had early multiple metastases to bone, liver, lymph nodes, and lungs. One explanation could be the localization of the tumor (Figure 1(a)). From both the MRI and surgical procedure, it is clear that this tumor had grown into the falx near the straight sinus. It is possible that the tumor spread hematogenously—perhaps even before surgery—due to this intimate contact with the venous structures outside the blood brain barrier. Other interesting observations in case 1 are strong and pronounced staining for p53, in both tumor and metastasis, and change of MGMT status from positive to negative when comparing primary tumor to metastasis (cut-off at 10%). The metastatic potential of the tumor cells increases with a gain-of-function mutation of p53 [12], and the negative MGMT status in the metastasis makes it less vulnerable to treatment with temozolomide [13]. This supports the idea of heterogeneity of the primary tumor and the idea of a subclone of temozolomide resistant cells managing to grow despite chemotherapy. These factors combined could have created an environment suitable for metastases. P53 gene mutations and differential clone selection have been suggested to be related to the metastatic potential of GBM [14]. In distant melanoma metastasis, it has been reported that one-third of the patients had MGMT hypermethylation [15]. It remains unknown if the interaction between MGMT status and temozolomide makes ECM from GBM more likely.

In the second case, the patient had two spontaneous bleeding incidents. These are rare in GBM compared to brain metastasis from malignant melanomas and renal cell carcinoma where bleeding incidents frequently occur. The GBM in this patient might have spread in relation to the intracerebral bleeding incidents but that is only theoretical, and in reality we do not have a plausible reason for spreading of this patient's GBM outside the blood brain barrier.

In conclusion, the cases presented might have hematogenous spreading in common as an explanation to extracranial metastases from GBM. Clinicians should keep in mind the potential of GBM to metastasize in order to diagnose ECM early. Even though this might not prolong the patients' survival, the quality of life and palliative treatment may improve. In the future, it would be relevant to make clinical guidelines to aid the clinicians in handling ECM in GBM patients.
